# Supplementary material for: Molecular Characterization and Identification of Potential Inhibitors for ‘E’ Protein of Dengue Virus
Source: Viruses. 2022 Apr 29;14(5):940. doi: 10.3390/v14050940 (PMC9143040; doi:10.3390/v14050940)

Dengue Typing Tool analysis results

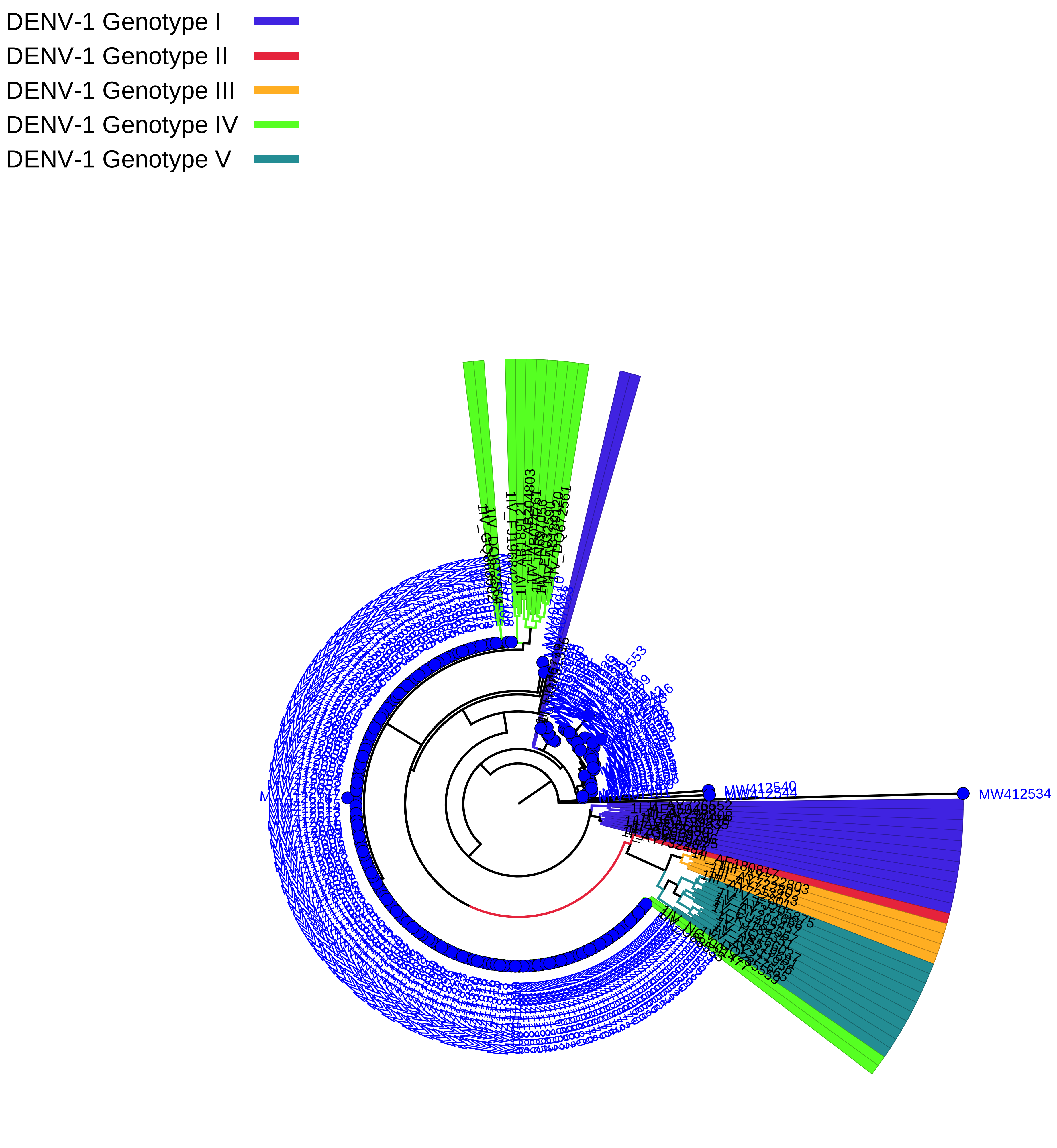

Supplement: Supplementary file 1 [file viruses-14-00940-s001.zip › viruses-1623514-supplementary/supplementary file 4_DENV4 phylogenetic tree.pdf]
