# Supplementary material for: Molecular Characterization and Identification of Potential Inhibitors for ‘E’ Protein of Dengue Virus
Source: Viruses. 2022 Apr 29;14(5):940. doi: 10.3390/v14050940 (PMC9143040; doi:10.3390/v14050940)
Supplement: Supplementary file 1 [file viruses-14-00940-s001.zip › viruses-1623514-supplementary/supplementary file 5_Xtra precision glide docking scores of 93 compounds.pdf]

Supplementary File S5. Xtra precision glide docking scores (kcal/mol) of 93 compounds against four dengue envelope protein serotypes

S.No.	Compound	DENV1	DENV2	DENV3	DENV4
1	Caricaxanthin	-3.122	-4.321	-3.312	-2.928
2	Violaxanthin	-4.363	-3.984	-4.061	-3.361
3	Zeaxanthin	-2.321	-4.310	-4.112	-3.987
4	Carpaine	-4.122	-5.014	-3.902	-2.976
5	Dehydrocarpaine I	-3.211	-4.451	-3.321	-2.212
6	Dehydrocarpaine II	-2.192	3.152	4.942	1.961
7	Cardenolide	-2.723	-1.549	-0.904	0.518
8	p-coumaric acid	-2.339	-4.231	-3.00	-3.108
**9**	**Chlorogenic acid**	-6.224	-4.273	**-8.723**	-6.79
10	Compound 6	-3.473	-2.878	-3.034	-1.468
11	Compound 1	0.315	4.312	7.548	4.321
12	Compound 2	-0.95	-1.152	2.411	3.4
13	3e	-4.419	-3.679	-2.976	-3.898
14	3h	2.121	1.342	-2.882	0.679
15	2-N-[3-Cyclopropyl-5-(tetrazol-1-yl)phenyl]-5-fluoro-4-N-(2,2,6,6-tetramethylpiperidin-4-yl)pyrimidine-2,4-diamine	3.121	-2.162	-1.141	-2.654
16	Choloroquine	-2.287	-3.127	-1.981	-4.327
17	Prednisolone	-5.201	-0.825	-4.574	-4.31
18	Lovastatin	-4.282	-3.283	-1.934	-3.268
19	Modipafant	-2.367	-3.901	-1.991	-0.708
20	Ketotifen	-2.684	-2.479	-1.998	-3.792
21	Balapiravir	-3.322	-1.984	-4.376	-3.069
22	Ribavirin	-6.013	-5.093	-6.013	-6.454
23	Celgosivir	-6.853	-4.598	-3.627	-4.925
24	UV-4	-2.321	-3.122	-2.451	-1.812
25	MON-6f-DGJ.	-5.75	1.246	0.751	-4.058
26	Ivermectin	-3.177	-1.211	-0.892	-1.752
27	Rolitetracycline	-5.702	0.148	-5.04	-5.069
28	Doxycycline	-5.798	-2.696	-6.987	-5.952
29	Sofosbuvir	-5.274	-3.842	-2.29	-5.813
30	Velpatasvir	-4.231	-3.581	1.875	0.089
31	Voxilaprevir	-2.624	0.04	-3.367	-2.234
32	Glecaprevir	-4.19	-1.49	-3.103	-2.124
33	Pibrentasvir	-2.512	-3.621	-4.215	-2.678
34	Amino{[(4S,5S)-4-amino-6,6,6-trifluoro-5-hydroxyhexyl]amino}methanimi nium	-1.324	-2.326	-3.124	-2.264
35	2,3,4,4,6-Pentamethoxychalcone	-3.542	-3.121	-3.816	-4.313
36	3,3,4,5-Tetrahydroxy-5-prenylbibenzyl	-3.621	-2.812	-3.042	-4.114
37	3,3,5-Trihydroxy-4-methoxy-5-prenylbibenzyl	-2.314	-3.141	-2.336	1.282
38	Aglycone	-4.395	-2.392	-3.315	-5.008
39	3-Acetoxy-4',5-dihydroxy-3'-prenyldihydrostilbene	-3.311	-4.213	-2.814	-3.121
40	4-O-Methylglycyrrhisoflavone	-5.611	-3.006	-3.248	-4.733
41	4-O-Methylneobavaisoflavone	-2.776	-4.632	-2.44	-4.206

42	8-prenylmucronulatol	-5.081	-2.771	-1.741	-4.371
43	**Agnuside**	**-7.881**	-4.71	-4.508	-6.532
44	Balsacone A	-5.02	-2.897	-4.747	-5.683
45	Balsacone B	-6.054	-2.96	-3.357	-5.454
46	Balsacone C	-5.024	-5.202	-4.37	-5.11
47	Chartaceone A2	-4.178	-3.825	-5.115	-2.203
48	Euchrestaflavanone A	-5.443	-4.237	-3.452	-4.217
49	Flemiflavanone D	-5.49	-4.753	-1.631	-5.518
50	Glycyrrhisoflavone	-4.162	-3.457	-2.821	-3.381
51	Isosilandrin A	-3.781	-2.872	-1.631	-2.886
52	Kanzonol Y	-6.68	-5.018	-5.195	-5.682
53	Licobenzofuran	-3.287	-2.181	-4.343	-3.144
54	Nishindaside	-4.859	-4.625	-3.807	-6.59
**55**	**Rhodiolin**	-4.489	**-5.1**	-5.262	-4.765
56	Silyhermin	-4.516	-4.318	-4.965	-2.431
57	Solophenol A	-7.551	-2.707	-4.401	-6.582
58	Umbelliprenin	-5.212	-4.813	-3.717	-2.833
59	β-octyl glucoside	-4.133	-3.611	-2.811	-2.214
60	1004361	-2.089	-1.19	2.374	-4.187
61	14272771	-2.409	-2.291	-1.774	-3.566
62	8900340	-4.067	-2.23	-1.332	-3.061
63	20610844	-6.912	-2.909	-1.898	-3.075
64	26124083	-2.691	-1.511	-0.605	-0.925
65	26124224	-2.984	-2.637	1.234	-2.34
66	A5	-3.501	-2.841	-1.297	-1.557
67	R1	-3.172	2.221	-4.781	-3.461
68	BOG	-2.63	-0.219	-2.817	0.077
**69**	**NITD448**	-5.503	-4.537	-3.227	**-6.889**
70	R2	-3.499	-2.287	-2.06	-3.708
71	100-1	-2.312	-2.268	-3.245	-1.874
72	100-22	-1.132	-3.321	-4.146	-2.411
73	148-6	-3.154	-2.814	-4.112	-3.198
74	GNF-2	-4.218	-3.255	-2.232	-1.187
75	LIGAND	-0.427	0.512	-2.184	-3.145
76	1	-1.812	-2.521	-1.332	-2.847
77	2	-2.874	-3.135	-2.14	-3.26
78	3	-4.569	-3.128	-3.213	-4.31
79	5	-3.857	-2.876	-4.18	-3.587
80	4	-3.992	-2.324	-3.187	-4.234
81	ARDP0006	-4.125	-3.153	-3.471	-2.849
82	PYRIMETHAMINE	-3.063	-0.987	-2.092	-3.116
83	ST-148	-4.179	-3.058	-2.141	-3.842
84	NICLOSAMIDE	-0.437	-3.862	-1.762	-3.581
85	M01	-3.646	-2.739	-3.595	-2.131
86	M02	-2.495	-3.299	-0.689	-0.476
87	P01	-4.524	-4.064	0.633	-3.252
88	P02	-2.117	-4.375	-1.816	-2.921
89	D01	-2.895	-2.84	-2.936	-0.689
90	D02	-2.636	-2.906	-1.545	-2.389
91	D03	-2.024	-3.72	-2.298	-2.756
92	D04	-2.84	-2.43	-2.12	-3.217
93	D05	-3.796	-2.018	-2.086	-2.687

The best docked existing inhibitors against four DENV serotypes are Agnuside for DENV1, Rhodiolin for DENV2, Chlorogenic acid for DENV3 and NITD448 for DENV4
