# Supplementary material for: Molecular Characterization and Identification of Potential Inhibitors for ‘E’ Protein of Dengue Virus
Source: Viruses. 2022 Apr 29;14(5):940. doi: 10.3390/v14050940 (PMC9143040; doi:10.3390/v14050940)
Supplement: Supplementary file 1 [file viruses-14-00940-s001.zip › viruses-1623514-supplementary/Supplementary file 6. Accession numbers of Dengue virus envelop (E) gene sequences.pdf]

Supplementary File S6. Accession numbers of Dengue virus envelop (E) gene sequences (two fragments; first-half (FH) and second-half (SH)) submitted in GenBank for each serotype.

Srl. No.	Sample no.	Sub-type	Serotype 1		Serotype 2		Serotype 3		Serotype 4	
			FH	SH	FH	SH	FH	SH	FH	SH
1	1	1	MW405216	MW411322	-	-	-	-	-	-
2	2	1	MW412526	MW411323	-	-	-	-	-	-
3	3	1	MW412527	MW411324	-	-	-	-	-	-
4	4	1	MW412528	MW411325	-	-	-	-	-	-
5	5	1	MW412529	MW411596	-	-	-	-	-	-
6	6	1	MW412530	MW411597	-	-	-	-	-	-
7	7	1	MW412531	MW411598	-	-	-	-	-	-
8	8	1	MW412532	MW411599	-	-	-	-	-	-
9	9	1	MW412533	MW411600	-	-	-	-	-	-
10	10	1	MW412534	MW411601	-	-	-	-	-	-
11	11	1	MW412535	MW411602	-	-	-	-	-	-
12	12	1	MW412536	MW411603	-	-	-	-	-	-
13	13	1	MW412537	MW411604	-	-	-	-	-	-
14	14	1	MW412538	MW411605	-	-	-	-	-	-
15	15	1	MW412539	MW411606	-	-	-	-	-	-
16	16	1	MW412540	MW411607	-	-	-	-	-	-
17	19	1	MW412541	MW411608	-	-	-	-	-	-
18	21	1	MW412542	MW411609	-	-	-	-	-	-
19	24	1	MW412543	MW411610	-	-	-	-	-	-
20	25	1	MW412544	MW411611	-	-	-	-	-	-
21	26	1	MW412545	MW411612	-	-	-	-	-	-

Supplementary File S6. Accession numbers of Dengue virus envelop (E) gene sequences (two fragments; first-half (FH) and second-half (SH)) submitted in GenBank for each serotype.

**Srl. No.**	**Sample no.**	**Sub-type**	**Serotype 1**		**Serotype 2**		**Serotype 3**		**Serotype 4**	
			**FH**	**SH**	**FH**	**SH**	**FH**	**SH**	**FH**	**SH**
22	27	1	MW412546	MW411613	-	-	-	-	-	-
23	28	1	MW412547	MW411614	-	-	-	-	-	-
24	29	1	MW412548	MW411615	-	-	-	-	-	-
25	30	1	MW412549	MW411616	-	-	-	-	-	-
26	33	1	MW412550	MW411617	-	-	-	-	-	-
27	34	1	MW412551	MW411618	-	-	-	-	-	-
28	35	1	MW412552	MW411619	-	-	-	-	-	-
29	39	1	MW412553	MW411620	-	-	-	-	-	-
30	40	1	MW412554	MW411621	-	-	-	-	-	-
31	41	1	MW412555	MW411622	-	-	-	-	-	-
32	43	1	MW412556	MW411623	-	-	-	-	-	-
33	46	1	MW407013	MW411624	-	-	-	-	-	-
34	47	1	MW407112	MW412594	-	-	-	-	-	-
35	48	1	MW407082	MW412595	-	-	-	-	-	-
36	49	1	MW407083	MW412596	-	-	-	-	-	-
37	51	1	MW407084	MW412597	-	-	-	-	-	-
38	52	1	MW407085	MW412598	-	-	-	-	-	-
39	53	1	MW407086	MW412599	-	-	-	-	-	-
40	54	1	MW407087	MW412600	-	-	-	-	-	-
41	56	1	MW407088	MW412601	-	-	-	-	-	-
42	57	1	MW407089	MW412602	-	-	-	-	-	-

Supplementary File S6. Accession numbers of Dengue virus envelop (E) gene sequences (two fragments; first-half (FH) and second-half (SH)) submitted in GenBank for each serotype.

Srl. No.	Sample no.	Sub-type	Serotype 1		Serotype 2		Serotype 3		Serotype 4	
			FH	SH	FH	SH	FH	SH	FH	SH
43	59	1	MW407090	MW412603	-	-	-	-	-	-
44	60	1	MW407091	MW412604	-	-	-	-	-	-
45	61	1	MW407092	MW412605	-	-	-	-	-	-
46	62	1	MW407093	MW412606	-	-	-	-	-	-
47	63	1	MW407094	MW412607	-	-	-	-	-	-
48	64	2	-	-	MW425355	MW425968	-	-	-	-
49	65	2	-	-	MW425356	MW425969	-	-	-	-
50	66	2	-	-	MW425357	MW425970	-	-	-	-
51	67	2	-	-	MW425358	MW425971	-	-	-	-
52	68	2	-	-	MW425359	MW425972	-	-	-	-
53	69	2	-	-	MW425360	MW425973	-	-	-	-
54	71	2	-	-	MW425361	MW425974	-	-	-	-
55	72	2	-	-	MW425362	MW425975	-	-	-	-
56	73	2	-	-	MW425363	MW425976	-	-	-	-
57	74	2	-	-	MW425364	MW425977	-	-	-	-
58	75	2	-	-	MW425365	MW425978	-	-	-	-
59	76	2	-	-	MW425366	MW425979	-	-	-	-
60	78	2	-	-	MW425367	MW425980	-	-	-	-
61	79	2	-	-	MW425368	MW425981	-	-	-	-
62	79	2	-	-	MW425369	MW425982	-	-	-	-
63	80	2	-	-	MW425370	MW425983	-	-	-	-

Supplementary File S6. Accession numbers of Dengue virus envelop (E) gene sequences (two fragments; first-half (FH) and second-half (SH)) submitted in GenBank for each serotype.

Srl. No.	Sample no.	Sub-type	Serotype 1		Serotype 2		Serotype 3		Serotype 4	
			FH	SH	FH	SH	FH	SH	FH	SH
64	81	2	-	-	MW425371	MW425984	-	-	-	-
65	82	2	-	-	MW425372	MW425985	-	-	-	-
66	83	2	-	-	MW425373	MW425986	-	-	-	-
67	84	2	-	-	MW425374	MW425987	-	-	-	-
68	85	2	-	-	MW425375	MW425988	-	-	-	-
69	86	2	-	-	MW425391	MW425989	-	-	-	-
70	87	2	-	-	MW425392	MW425991	-	-	-	-
71	89	2	-	-	MW425393	MW425992	-	-	-	-
72	91	2	-	-	MW425394	MW425993	-	-	-	-
73	92	2	-	-	MW425395	MW425994	-	-	-	-
74	93	2	-	-	MW425396	MW425995	-	-	-	-
75	94	2	-	-	MW425397	MW425996	-	-	-	-
76	95	2	-	-	MW425398	MW425997	-	-	-	-
77	96	2	-	-	MW425399	MW425998	-	-	-	-
78	97	2	-	-	MW425400	MW425999	-	-	-	-
79	98	2	-	-	MW425401	MW426000	-	-	-	-
80	99	2	-	-	MW425402	MW426001	-	-	-	-
81	101	2	-	-	MW425403	MW426002	-	-	-	-
82	102	2	-	-	MW425404	MW426003	-	-	-	-
83	103	2	-	-	MW425405	MW426004	-	-	-	-
84	104	2	-	-	MW425406	MW426005	-	-	-	-

Supplementary File S6. Accession numbers of Dengue virus envelop (E) gene sequences (two fragments; first-half (FH) and second-half (SH)) submitted in GenBank for each serotype.

Srl. No.	Sample no.	Sub-type	Serotype 1		Serotype 2		Serotype 3		Serotype 4	
			FH	SH	FH	SH	FH	SH	FH	SH
85	105	2	-	-	MW425407	MW426006	-	-	-	-
86	106	2	-	-	MW425408	MW426007	-	-	-	-
87	107	1	MW407095	MW412608	-	-	-	-	-	-
88	108	1	MW407096	MW412609	-	-	-	-	-	-
89	109	1	MW407097	MW412610	-	-	-	-	-	-
90	110	2	-	-	MW425409	MW426008	-	-	-	-
91	111	2	-	-	MW425410	MW426009	-	-	-	-
92	113	2	-	-	MW425411	MW426010	-	-	-	-
93	114	2	-	-	MW425519	MW426011	-	-	-	-
94	118	2	-	-	MW425520	MW426012	-	-	-	-
95	119	2	-	-	MW425521	MW426013	-	-	-	-
96	120	2	-	-	MW425522	MW426014	-	-	-	-
97	121	2	-	-	MW425523	MW426015	-	-	-	-
98	122	2	-	-	MW425524	MW426016	-	-	-	-
99	123	2	-	-	MW425525	MW426017	-	-	-	-
100	125	2	-	-	MW425526	MW426018	-	-	-	-
101	126	2	-	-	MW425527	MW426019	-	-	-	-
102	127	2	-	-	MW425528	MW426020	-	-	-	-
103	129	2	-	-	MW425529	MW426021	-	-	-	-
104	130	2	-	-	MW425530	MW426022	-	-	-	-
105	131	2	-	-	MW425531	MW426023	-	-	-	-

Supplementary File S6. Accession numbers of Dengue virus envelop (E) gene sequences (two fragments; first-half (FH) and second-half (SH)) submitted in GenBank for each serotype.

Srl. No.	Sample no.	Sub-type	Serotype 1		Serotype 2		Serotype 3		Serotype 4	
			FH	SH	FH	SH	FH	SH	FH	SH
106	132	2	-	-	MW425532	MW426024	-	-	-	-
107	133	2	-	-	MW425533	MW426025	-	-	-	-
108	134	2	-	-	MW425534	MW426026	-	-	-	-
109	135	2	-	-	MW425535	MW426027	-	-	-	-
110	136	2	-	-	MW425536	MW426028	-	-	-	-
111	137	2	-	-	MW425537	MW426029	-	-	-	-
112	138	2	-	-	MW425538	MW426030	-	-	-	-
113	140	2	-	-	MW425539	MW426031	-	-	-	-
114	141	2	-	-	MW425542	MW426032	-	-	-	-
115	142	2	-	-	MW425543	MW426033	-	-	-	-
116	143	2	-	-	MW425544	MW426034	-	-	-	-
117	144	2	-	-	MW425545	MW426035	-	-	-	-
118	145	2	-	-	MW425546	MW426036	-	-	-	-
119	146	2	-	-	MW425547	MW426037	-	-	-	-
120	147	2	-	-	MW425548	MW426038	-	-	-	-
121	150	2	-	-	MW425549	MW426039	-	-	-	-
122	151	2	-	-	MW425550	MW426040	-	-	-	-
123	152	2	-	-	MW425551	MW426041	-	-	-	-
124	153	2	-	-	MW425552	MW426042	-	-	-	-
125	154	2	-	-	MW425553	MW426043	-	-	-	-
126	155	2	-	-	MW425554	MW426044	-	-	-	-

Supplementary File S6. Accession numbers of Dengue virus envelop (E) gene sequences (two fragments; first-half (FH) and second-half (SH)) submitted in GenBank for each serotype.

Srl. No.	Sample no.	Sub-type	Serotype 1		Serotype 2		Serotype 3		Serotype 4	
			FH	SH	FH	SH	FH	SH	FH	SH
127	156	2	-	-	MW425555	MW426076	-	-	-	-
128	157	2	-	-	MW425556	MW426077	-	-	-	-
129	158	2	-	-	MW425557	MW426078	-	-	-	-
130	159	2	-	-	MW425558	MW426079	-	-	-	-
131	160	2	-	-	MW425559	MW426080	-	-	-	-
132	162	2	-	-	MW425560	MW426081	-	-	-	-
133	164	2	-	-	MW425561	MW426082	-	-	-	-
134	165	2	-	-	MW425562	MW426083	-	-	-	-
135	166	2	-	-	MW425753	MW426084	-	-	-	-
136	167	2	-	-	MW425754	MW426085	-	-	-	-
137	169	2	-	-	MW425755	MW426086	-	-	-	-
138	170	2	-	-	MW425756	MW426087	-	-	-	-
139	171	2	-	-	MW425757	MW426088	-	-	-	-
140	174	2	-	-	MW425758	MW426089	-	-	-	-
141	176	2	-	-	MW425759	MW426090	-	-	-	-
142	177	2	-	-	MW425760	MW426091	-	-	-	-
143	178	2	-	-	MW425761	MW426092	-	-	-	-
144	179	2	-	-	MW425762	MW426093	-	-	-	-
145	180	2	-	-	MW425763	MW426094	-	-	-	-
146	182	2	-	-	MW425764	MW426095	-	-	-	-
147	183	2	-	-	MW425765	MW426096	-	-	-	-

Supplementary File S6. Accession numbers of Dengue virus envelop (E) gene sequences (two fragments; first-half (FH) and second-half (SH)) submitted in GenBank for each serotype.

Srl. No.	Sample no.	Sub-type	Serotype 1		Serotype 2		Serotype 3		Serotype 4	
			FH	SH	FH	SH	FH	SH	FH	SH
148	185	2	-	-	MW425766	MW426097	-	-	-	-
149	186	2	-	-	MW425767	MW426098	-	-	-	-
150	187	2	-	-	MW425768	MW426099	-	-	-	-
151	188	2	-	-	MW425769	MW426100	-	-	-	-
152	189	2	-	-	MW425770	MW426101	-	-	-	-
153	190	2	-	-	MW425771	MW426102	-	-	-	-
154	191	2	-	-	MW425772	MW426103	-	-	-	-
155	192	2	-	-	MW425773	MW426104	-	-	-	-
156	193	2	-	-	MW425774	MW426105	-	-	-	-
157	195	2	-	-	MW425775	MW426106	-	-	-	-
158	196	2	-	-	MW425776	MW426107	-	-	-	-
159	197	2	-	-	MW425777	MW426108	-	-	-	-
160	198	2	-	-	MW425778	MW426109	-	-	-	-
161	199	2	-	-	MW425779	MW426110	-	-	-	-
162	201	2	-	-	MW425780	MW426111	-	-	-	-
163	202	2	-	-	MW425781	MW426112	-	-	-	-
164	203	2	-	-	MW425782	MW426113	-	-	-	-
165	204	2	-	-	MW425783	MW426114	-	-	-	-
166	205	2	-	-	MW425784	MW426115	-	-	-	-
167	206	2	-	-	MW425785	MW426116	-	-	-	-
168	207	2	-	-	MW425786	MW426117	-	-	-	-

Supplementary File S6. Accession numbers of Dengue virus envelop (E) gene sequences (two fragments; first-half (FH) and second-half (SH)) submitted in GenBank for each serotype.

Srl. No.	Sample no.	Sub-type	Serotype 1		Serotype 2		Serotype 3		Serotype 4	
			FH	SH	FH	SH	FH	SH	FH	SH
169	208	2	-	-	MW425787	MW426118	-	-	-	-
170	209	2	-	-	MW425788	MW426119	-	-	-	-
171	210	2	-	-	MW425789	MW426120	-	-	-	-
172	211	2	-	-	MW425790	MW426121	-	-	-	-
173	212	2	-	-	MW425791	MW426122	-	-	-	-
174	213	2	-	-	MW425792	MW426123	-	-	-	-
175	214	2	-	-	MW425793	MW426124	-	-	-	-
176	215	2	-	-	MW425794	MW426125	-	-	-	-
177	216	2	-	-	MW425795	MW426126	-	-	-	-
178	217	2	-	-	MW425796	MW426127	-	-	-	-
179	218	2	-	-	MW425797	MW426128	-	-	-	-
180	219	2	-	-	MW425798	MW426129	-	-	-	-
181	220	2	-	-	MW425799	MW426130	-	-	-	-
182	221	2	-	-	MW425800	MW426131	-	-	-	-
183	222	2	-	-	MW425801	MW426132	-	-	-	-
184	223	2	-	-	MW425802	MW426133	-	-	-	-
185	224	2	-	-	MW425803	MW426134	-	-	-	-
186	225	2	-	-	MW425804	MW426135	-	-	-	-
187	226	2	-	-	MW425805	MW426136	-	-	-	-
188	227	2	-	-	MW425806	MW426137	-	-	-	-
189	228	2	-	-	MW425807	MW426138	-	-	-	-

Supplementary File S6. Accession numbers of Dengue virus envelop (E) gene sequences (two fragments; first-half (FH) and second-half (SH)) submitted in GenBank for each serotype.

Srl. No.	Sample no.	Sub-type	Serotype 1		Serotype 2		Serotype 3		Serotype 4	
			FH	SH	FH	SH	FH	SH	FH	SH
190	229	2	-	-	MW425808	MW426139	-	-	-	-
191	230	3	-	-	-	-	MW413419	MW418135		
192	231	3	-	-	-	-	MW413420	MW418136		
193	232	3	-	-	-	-	MW413421	MW418137		
194	234	3	-	-	-	-	MW413422	MW418138		
195	235	3	-	-	-	-	MW413423	MW418139		
196	237	3	-	-	-	-	MW413424	MW418140		
197	239	3	-	-	-	-	MW413425	MW418141		
198	240	3	-	-	-	-	MW413426	MW418142		
199	241	3	-	-	-	-	MW413427	MW418143		
200	242	3	-	-	-	-	MW413428	MW418144		
201	244	3	-	-	-	-	MW413429	MW418145		
202	245	3	-	-	-	-	MW413430	MW418146		
203	246	3	-	-	-	-	MW413431	MW418147		
204	247	3	-	-	-	-	MW413432	MW418148		
205	248	3	-	-	-	-	MW413433	MW418149		
206	249	3	-	-	-	-	MW413434	MW418150		
207	251	3	-	-	-	-	MW414870	MW418182		
208	252	3	-	-	-	-	MW414871	MW418183		
209	253	3	-	-	-	-	MW414872	MW418184		
210	254	3	-	-	-	-	MW414873	MW418185		

Supplementary File S6. Accession numbers of Dengue virus envelop (E) gene sequences (two fragments; first-half (FH) and second-half (SH)) submitted in GenBank for each serotype.

Srl. No.	Sample no.	Sub-type	Serotype 1		Serotype 2		Serotype 3		Serotype 4	
			FH	SH	FH	SH	FH	SH	FH	SH
211	255	3	-	-	-	-	MW414874	MW418186		
212	256	3	-	-	-	-	MW414875	MW418187		
213	257	3	-	-	-	-	MW414876	MW418188		
214	258	3	-	-	-	-	MW414877	MW418189		
215	259	3	-	-	-	-	MW414878	MW418190		
216	261	3	-	-	-	-	MW414879	MW418470		
217	262	3	-	-	-	-	MW414880	MW418471		
218	263	3	-	-	-	-	MW414881	MW418472		
219	264	3	-	-	-	-	MW414882	MW418473		
220	266	3	-	-	-	-	MW414883	MW418474		
221	267	4	-	-	-	-	-	-	MW419301	MW423386
222	268	4	-	-	-	-	-	-	MW419302	MW423387
223	269	4	-	-	-	-	-	-	MW419303	MW423388
224	270	4	-	-	-	-	-	-	MW419304	MW423389
225	271	4	-	-	-	-	-	-	MW419305	MW423390
226	272	4	-	-	-	-	-	-	MW419306	MW423391
227	273	4	-	-	-	-	-	-	MW419307	MW423392
228	274	4	-	-	-	-	-	-	MW419308	MW423393
229	275	4	-	-	-	-	-	-	MW419309	MW423394
230	276	4	-	-	-	-	-	-	MW419310	MW423395
231	277	4	-	-	-	-	-	-	MW419311	MW423396

Supplementary File S6. Accession numbers of Dengue virus envelop (E) gene sequences (two fragments; first-half (FH) and second-half (SH)) submitted in GenBank for each serotype.

Srl. No.	Sample no.	Sub-type	Serotype 1		Serotype 2		Serotype 3		Serotype 4	
			FH	SH	FH	SH	FH	SH	FH	SH
232	278	4	-	-	-	-	-	-	MW419312	MW423397
233	279	4	-	-	-	-	-	-	MW419313	MW423398
234	280	4	-	-	-	-	-	-	MW419314	MW423399
235	281	4	-	-	-	-	-	-	MW419315	MW423400
236	282	4	-	-	-	-	-	-	MW419316	MW423401
237	283	4	-	-	-	-	-	-	MW421410	MW423582
238	284	4	-	-	-	-	-	-	MW421411	MW423583
239	285	4	-	-	-	-	-	-	MW421412	MW423584
240	286	4	-	-	-	-	-	-	MW421413	MW423585
241	287	4	-	-	-	-	-	-	MW421414	MW423586
242	288	4	-	-	-	-	-	-	MW421415	MW423587
243	289	4	-	-	-	-	-	-	MW421416	MW423588
244	290	4	-	-	-	-	-	-	MW421417	MW423589
245	291	4	-	-	-	-	-	-	MW421418	MW423590
246	292	4	-	-	-	-	-	-	MW421419	MW423591
247	293	4	-	-	-	-	-	-	MW421420	MW423592
248	294	4	-	-	-	-	-	-	MW421421	MW423593
249	295	4	-	-	-	-	-	-	MW421422	MW423594
250	296	4	-	-	-	-	-	-	MW421423	MW423595
251	297	4	-	-	-	-	-	-	MW421424	MW423596
252	298	4	-	-	-	-	-	-	MW421425	MW423597

Supplementary File S6. Accession numbers of Dengue virus envelop (E) gene sequences (two fragments; first-half (FH) and second-half (SH)) submitted in GenBank for each serotype.

Srl. No.	Sample no.	Sub-type	Serotype 1		Serotype 2		Serotype 3		Serotype 4	
			FH	SH	FH	SH	FH	SH	FH	SH
253	299	4	-	-	-	-	-	-	MW421740	MW423598
254	300	4	-	-	-	-	-	-	MW421741	MW423599
255	301	4	-	-	-	-	-	-	MW421742	MW423600
256	302	4	-	-	-	-	-	-	MW421743	MW423601
257	303	4	-	-	-	-	-	-	MW421744	MW423936
258	304	4	-	-	-	-	-	-	MW421745	MW423937
259	305	4	-	-	-	-	-	-	MW421746	MW423938
260	306	4	-	-	-	-	-	-	MW421747	MW423939
261	307	4	-	-	-	-	-	-	MW421748	MW423940
262	308	4	-	-	-	-	-	-	MW421749	MW423941
263	309	4	-	-	-	-	-	-	MW421750	MW423942
264	310	4	-	-	-	-	-	-	MW421751	MW423943
265	311	4	-	-	-	-	-	-	MW422735	MW423944
266	312	4	-	-	-	-	-	-	MW422736	MW423945
267	313	4	-	-	-	-	-	-	MW422737	MW423946
268	314	4	-	-	-	-	-	-	MW422738	MW423947
269	316	4	-	-	-	-	-	-	MW422739	MW423948
270	317	4	-	-	-	-	-	-	MW422740	MW423949
271	318	4	-	-	-	-	-	-	MW422741	MW423950
272	319	4	-	-	-	-	-	-	MW422742	MW423951
273	320	4	-	-	-	-	-	-	MW422743	MW423952

Supplementary File S6. Accession numbers of Dengue virus envelop (E) gene sequences (two fragments; first-half (FH) and second-half (SH)) submitted in GenBank for each serotype.

Srl. No.	Sample no.	Sub-type	Serotype 1		Serotype 2		Serotype 3		Serotype 4	
			FH	SH	FH	SH	FH	SH	FH	SH
274	321	4	-	-	-	-	-	-	MW422744	MW423953
275	322	4	-	-	-	-	-	-	MW422745	MW423954
276	323	4	-	-	-	-	-	-	MW422746	MW423955
277	324	4	-	-	-	-	-	-	MW422747	MW423958
278	325	4	-	-	-	-	-	-	MW422748	MW423959
279	326	4	-	-	-	-	-	-	MW422749	MW423960
280	328	4	-	-	-	-	-	-	MW422750	MW423961
281	329	4	-	-	-	-	-	-	MW422790	MW423962
282	330	4	-	-	-	-	-	-	MW422791	MW423963
283	331	4	-	-	-	-	-	-	MW422792	MW423964
284	332	4	-	-	-	-	-	-	MW422793	MW423965
285	333	4	-	-	-	-	-	-	MW422794	MW423966
286	334	4	-	-	-	-	-	-	MW422795	MW423967
287	335	4	-	-	-	-	-	-	MW422796	MW423968
288	336	4	-	-	-	-	-	-	MW422797	MW423969
289	337	4	-	-	-	-	-	-	MW422798	MW423970
290	338	1,2	MW407098	MW412611	MW425809	MW426140	-	-	-	-
291	340	1,2	MW407099	MW412612	MW425810	MW426141	-	-	-	-
292	341	1,2	MW407100	MW412613	MW425811	MW426142	-	-	-	-
293	342	1,2	MW407101	MW412614	MW425812	MW426143	-	-	-	-
294	343	1,2	MW407102	MW412615	MW425813	MW426148	-	-	-	-

Supplementary File S6. Accession numbers of Dengue virus envelop (E) gene sequences (two fragments; first-half (FH) and second-half (SH)) submitted in GenBank for each serotype.

Srl. No.	Sample no.	Sub-type	Serotype 1		Serotype 2		Serotype 3		Serotype 4	
			FH	SH	FH	SH	FH	SH	FH	SH
295	344	1,2	MW407103	MW412616	MW425814	MW426149	-	-	-	-
296	345	1,2	MW407104	MW412617	MW425815	MW426150	-	-	-	-
297	346	1,2	MW407105	MW412651	MW425816	MW426151	-	-	-	-
298	347	1,2	MW407106	MW412652	MW425817	MW426152	-	-	-	-
299	348	1,2	MW407107	MW412653	MW425818	MW426153	-	-	-	-
300	349	1,2	MW407108	MW412654	MW425819	MW426154	-	-	-	-
301	350	1,2	MW407109	MW412655	MW425820	MW426155	-	-	-	-
302	351	1,2	MW407110	MW412656	MW425821	MW426156	-	-	-	-
303	352	1,2	MW407111	MW412657	MW425822	MW426157	-	-	-	-
304	353	1,2	MW406990	MW412658	MW425823	MW426158	-	-	-	-
305	354	1,2	MW410953	MW412659	MW425824	MW426159	-	-	-	-
306	355	1,2	MW410954	MW412660	MW425825	MW426160	-	-	-	-
307	356	1,2	MW410955	MW412661	MW425826	MW426161	-	-	-	-
308	357	1,2	MW410956	MW412662	MW425827	MW426162	-	-	-	-
309	358	1,2	MW410957	MW412663	MW425828	MW426163	-	-	-	-
310	359	1,2	MW410958	MW412664	MW425829	MW426164	-	-	-	-
311	360	1,2	MW410959	MW412665	MW425830	MW426165	-	-	-	-
312	361	1,2	MW410960	MW412666	MW425831	MW426166	-	-	-	-
313	362	1,2	MW410961	MW412667	MW425832	MW426167	-	-	-	-
314	363	1,2,3	MW410962	MW412668	MW425833	MW426168	MW414884	MW418475	-	-
315	364	1,2,3	MW410963	MW412669	MW425834	MW426169	MW414885	MW418476	-	-

Supplementary File S6. Accession numbers of Dengue virus envelop (E) gene sequences (two fragments; first-half (FH) and second-half (SH)) submitted in GenBank for each serotype.

Srl. No.	Sample no.	Sub-type	Serotype 1		Serotype 2		Serotype 3		Serotype 4	
			FH	SH	FH	SH	FH	SH	FH	SH
316	365	1,2,3	MW410964	MW412670	MW425835	MW426170	MW415410	MW418477	-	-
317	366	1,2,3	MW410965	MW412671	MW425836	MW426171	MW415411	MW418478	-	-
318	367	1,2,3,4	MW410966	MW412672	MW425944	MW426172	MW418039	MW418479	MW422799	MW423971
319	368	1,2,3,4	MW410967	MW412673	MW425945	MW426173	MW418040	MW418480	MW422800	MW423972
320	369	1,2,3,4	MW410968	MW412674	MW425946	MW426174	MW418041	MW418481	MW422801	MW423973
321	370	1,2,3,4	MW410969	MW412941	MW425947	MW426175	MW418042	MW418482	MW422802	MW423974
322	371	1,2,3,4	MW410970	MW412942	MW425948	MW426176	MW418043	MW418483	MW422803	MW423975
323	372	1,2,3,4	MW410971	MW412943	MW425949	MW426177	MW418044	MW418484	MW422804	MW423976
324	373	1,2,3,4	MW410972	MW412944	MW425950	MW426178	MW418045	MW418485	MW422805	MW423977
325	374	1,2,3,4	MW410973	MW412945	MW425951	MW426179	MW418046	MW418486	MW422837	MW424374
326	375	1,2,3,4	MW410974	MW412946	MW425952	MW426180	MW418047	MW418487	MW422838	MW424375
327	376	1,2,3,4	MW410975	MW412947	MW425953	MW426181	MW418048	MW418488	MW422839	MW424376
328	377	1,2,4	MW410976	MW412948	MW425954	MW426182	-	-	MW422840	MW424377
329	378	1,2,4	MW410977	MW412949	MW425955	MW426183	-	-	MW422841	MW424378
330	379	1,3	MW410978	MW412950	-	-	MW418049	MW418489	-	-
331	380	1,3	MW410979	MW412951	-	-	MW418050	MW418490	-	-
332	381	1,3,4	MW410980	MW412952	-	-	MW418051	MW418491	MW422842	MW424379
333	382	1,3,4	MW410981	MW412953	-	-	MW418052	MW418492	MW422843	MW424380
334	383	1,3,4	MW410982	MW412954	-	-	MW418053	MW418493	MW422844	MW424381
335	384	1,3,4	MW410983	MW412955	-	-	MW418054	MW418494	MW422845	MW424382
336	385	1,4	MW411302	MW412956	-	-	-	-	MW422846	MW424383

Supplementary File S6. Accession numbers of Dengue virus envelop (E) gene sequences (two fragments; first-half (FH) and second-half (SH)) submitted in GenBank for each serotype.

Srl. No.	Sample no.	Sub-type	Serotype 1		Serotype 2		Serotype 3		Serotype 4	
			FH	SH	FH	SH	FH	SH	FH	SH
337	386	1,4	MW411303	MW412957	-	-	-	-	MW422847	MW424384
338	387	1,4	MW411304	MW412958	-	-	-	-	MW422848	MW424385
339	388	1,4	MW411305	MW412959	-	-	-	-	MW422849	MW424386
340	389	1,4	MW411306	MW412960	-	-	-	-	MW422850	MW424387
341	390	1,4	MW411307	MW412961	-	-	-	-	MW422851	MW424388
342	391	2,3	-	-	MW425956	MW426184	MW418055	MW418495	-	-
343	392	2,3	-	-	MW425957	MW426185	MW418056	MW418496	-	-
344	393	2,3	-	-	MW425958	MW426186	MW418057	MW418497	-	-
345	394	2,3	-	-	MW425959	MW426187	MW418058	MW418498	-	-
346	395	2,3	-	-	MW425960	MW426188	MW418059	MW418499	-	-
347	396	2,3	-	-	MW425961	MW426189	MW418060	MW418500	-	-
348	397	2,3	-	-	MW425962	MW426190	MW418061	MW418501	-	-
349	398	2,3	-	-	MW425963	MW426191	MW418062	MW418502	-	-
350	399	2,4	MW425964	MW426192	-	-	-	-	MW422852	MW424389
351	400	2,4	MW425965	MW426193	-	-	-	-	MW423382	MW424390
352	401	2,4	MW425966	MW426194	-	-	-	-	MW423383	MW424391
353	402	3,4	-	-	-	-	MW418063	MW418503	MW423384	MW424392
354	403	3,4	-	-	-	-	MW418064	MW418504	MW423385	MW424393
